# Cook like a Boss Online: an adapted intervention during the COVID-19 pandemic that effectively improved children’s perceived cooking competence, movement competence and wellbeing

**DOI:** 10.1186/s12966-022-01378-x

**Published:** 2022-12-09

**Authors:** Lynsey Hollywood, Johann Issartel, David Gaul, Amanda McCloat, Elaine Mooney, Clare Elizabeth Collins, Fiona Lavelle

**Affiliations:** 1 Department of Hospitality and Tourism Management, Ulster University Business School, Ulster University, Belfast, UK; 2 Multisensory Motor Learning Lab, School of Health and Human Performance, Dublin City University, Dublin, Ireland; 3 Department of Business, Technological University Dublin, Dublin, Ireland; 4 School of Home Economics, National Centre for Excellence for Home Economics, St. Angela’s College Sligo, Sligo, Ireland; 5 School of Health Sciences, College of Health, Medicine and Wellbeing, The University of Newcastle, 2308 Callaghan, NSW Australia; 6 Hunter Medical Research Institute, 2305 New Lambton Heights, NSW Australia; 7 Institute for Global Food Security, School of Biological Sciences, Queen’s University Belfast, 19 Chlorine Gardens, BT9 5DL Belfast, UK; 8 Department of Nutritional Sciences, School of Life Course & Population Sciences, King’s College London, London, UK

**Keywords:** e-health, Cooking, Movement, Wellbeing, COVID-19, Children, Intervention, Perceived competence, Culinary nutrition, Motor skills

## Abstract

**Background:**

The COVID-19 pandemic has further exacerbated physical inactivity, poor dietary intake and reduced mental wellbeing, contributing factors to non-communicable diseases in children. Cooking interventions are proposed as having a positive influence on children’s diet quality. Motor skills have been highlighted as essential for performance of cooking skills, and this movement may contribute to wellbeing. Additionally, perceived competence is a motivator for behaviour performance and thus important for understanding intervention effectiveness. Therefore, this research aimed to assess the effectiveness of an adapted virtual theory-based cooking intervention on perceived cooking competence, perceived movement competence and wellbeing.

**Methods:**

The effective theory-driven and co-created ‘Cook Like A Boss’ was adapted to a virtual five day camp-styled intervention, with 248 children across the island of Ireland participating during the pandemic. Pre- and post-intervention assessments of perceived cooking competence, perceived movement competence and wellbeing using validated measurements were completed through online surveys. Bivariate Correlations, paired samples t-tests and Hierarchical multiple regression modelling was conducted using SPSS to understand the relationships between the variables and the effect of the intervention.

**Results:**

210 participants had matched survey data and were included in analysis. Significant positive correlations were shown between perceived cooking competence, perceived movement competence and wellbeing (*P* < 0.05). Children’s perceived cooking competence (*P* < 0.001, medium to large effect size), perceived movement competence (*P* < 0.001, small to medium effect size) and wellbeing (*P* = 0.013, small effect size) all significantly increased from pre to post intervention. For the Hierarchical regression, the final model explained 57% of the total variance in participants’ post-intervention perceived cooking competence. Each model explained a significant amount of variance (*P* < 0.05). Pre-intervention perceived cooking competence, wellbeing, age and perceived movement competence were significant predictors for post-intervention perceived cooking competence in the final model.

**Conclusion:**

The ‘Cook Like A Boss’ Online intervention was an adapted virtual outreach intervention. It provides initial evidence for the associations between perceived cooking competence, perceived movement and wellbeing as well as being effective in their improvement. This research shows the potential for cooking to be used as a mechanism for targeting improvements in not only diet quality but also movement and wellbeing.

**Trial Registration:**

NCT05395234. Retrospectively registered on 26th May 2022.

**Supplementary Information:**

The online version contains supplementary material available at 10.1186/s12966-022-01378-x.

## Background

### Child health and the COVID-19 pandemic

The global prevalence of childhood obesity, a multifactorial chronic condition, is a major public health concern and can have a detrimental impact on a child’s physical health and mental wellbeing [1, 2]. Alarmingly, this has paralled rises in other non-communicable diseases in children, such as cardiovascular diseases and diabetes [3, 4]. Some of the reported contributing factors, such as physical inactivity, poor dietary intake and reduced mental wellbeing, have been further exacerbated during the recent COVID-19 pandemic [5–9]. Furthermore, changes to individual’s dietary intakes may have fluctuated during the pandemic, including increases in both saturated fat, and fruit and vegetable intakes [10]. In addition, more frequent home cooking behaviours, including an increase in child participation in cooking and baking activities were reported [10, 11]. This may be considered a positive, as the decline in cooking and food preparation skills has been attributed to the rise of obesity [12]. Prior to the pandemic, there has been a lack of child involvement in cooking activities in the home environment due to parental time pressures and safety concerns [13]. This missed opportunity may have had negative consequences as research shows that learning cooking skills at younger ages is associated with positive dietary outcomes in adulthood and that these skills are retained to adulthood [14, 15]. In addition, children’s cooking interventions have the potential to influence their food related preferences, attitudes, enjoyment, behaviours and self-efficacy [16–18].

### Motor skills underpinning cooking skills and movement and potential contribution to wellbeing

The importance of children’s gross and fine motor skills to enable the performance of cooking skills has been previously highlighted [19]. For example, fine motor skills are required to chop ingredients and gross motor skills are needed for stirring [19]. These are also essential skills needed to take part in activities of daily living and physical activity [20, 21]. Additionally, children’s perceived movement competence contributes to their physical self-efficacy and in turn, their physical activity levels [22]. Furthermore, movement and physical activity has been associated with positive mental wellbeing [23, 24]. More recently, cooking has been associated with positive wellbeing in adults and adolescents [25, 26], with the physical movements required to perform the cooking skills suggested as a possible mechanism for this improvement in wellbeing [27].

### Cooking interventions and outcomes

Children’s cooking interventions have been highlighted as potential positive mechanisms for influencing children’s diet quality [16–18], with successful elements being considered as sessions led by a culinary professional [28, 29], a greater number of sessions [30], and more than one session a week [16, 17]. However, methodological issues remain [18]. Design weaknesses include a lack of underpinning theory, model and/or framework for their development, a lack of control groups and sample size calculations [16, 18]. Furthermore, there is limited use of validated measurement tools for the assessment of the intervention effectiveness. Perceived competence, an individual’s self-efficacy for undertaking a specific task, is a greater motivator to perform a behaviour than actual competence [31], and an important element for assessing the effectiveness of interventions.

### Aim and hypotheses

Therefore, this research aimed to assess the effectiveness of an adapted virtual theory-based cooking intervention (Cook Like A Boss Online) on perceived cooking competence, perceived movement competence and wellbeing, using validated measures. A secondary aim was to explore the relationships between these variables.

We hypothesised that there would be a general positive relationship between perceived cooking competence, perceived movement competence and wellbeing (investigated through pre-intervention variables) (H1). We expected that there would be a significant increase in perceived cooking competence, movement competence and wellbeing post intervention (H2). In addition, we hypothesised that demographic factors (age, gender, prior cooking experience) as well as both pre and post intervention perceived motor competence and wellbeing would predict post intervention perceived cooking competence.

## Methods

### Participants and ethical approval

Due to the COVID-19 pandemic, all aspects of this intervention were conducted online, and it was designed as an outreach/supportive programme for parents and children during a difficult time period. Parents of potential participants were recruited through researcher networks and social media channels. Upon contacting the research team, parents were provided with further information on the programme and the links to both the parent and child pre-intervention surveys (parental data not shown). At initiation of the parental survey, parents were presented with additional information on the research study and provided consent. Parents were made aware that their children were not obliged to take part and that they could withdraw their data or their children’s data at any time point up to data analysis, without reason or consequence. Participants were eligible if they were aged between 9 and 12 years and had not progressed to second level school. The camp was provided free to all participants and parents were provided with a £/€20 voucher upon completion of all four surveys as a contribution towards the cost of ingredients, in line with the original camp where ingredients were provided. The research was conducted in accordance with the Declaration of Helsinki, and as such, in light of the circumstances, it was decided that it was unethical to have a waitlist (delayed intervention) control group. As the intervention was designed to be conducted over a school holiday period (Easter holidays) and ran for a week, a delayed group would have to wait a number of months to take part. Due to the uncertainty of the pandemic situation and the need of parents for activities for children, all eligible participants up to the maximum capacity took part. Ethical approval was received from the Faculty of Medicine, Health and Life Sciences Research Ethics Committee, Queen’s University Belfast (MHLS 21_24).

### Intervention design, structure and content

The intervention was adapted from the original ‘Cook Like A Boss’ camp intervention [32], which was developed in line with the Cook-Ed model [33] and theoretically underpinned by Social Learning theory (SLT) and Experiential Learning Theory (ELT) [34, 35]. The camp was designed to introduce the children to a range of food and skills and to nurture an initial interest in cooking. The original age-appropriate co-created content was used for the intervention [19, 32] with minor adaptions. These included the removal of making pasta from scratch due to equipment concerns, reordering days (changing the order of the days from the order in the original camp), and chef suggestions around alternative household equipment/ingredients to use. The adaptions were conducted to promote and ensure inclusivity of the programme to as many participants, i.e., removal/alternative equipment suggestions. The chef who facilitated the ‘Cook Like A Boss’ camp recorded the video version of the camp. He had over 30 years’ experience including running cooking classes for children. The adapted online version of the camp included five daily videos of the chef performing the recipes that were emailed to the participant’s parents prior to the session. The video recipes could be completed at any time during the day that suited the family. The five daily videos and how they were adapted from the original programme are presented in Table 1. Additionally, as the camp was virtual, to generate some social connectedness for the participants that would have naturally occurred in the original camp, parents were encouraged to share progress pictures (different stages in the recipe preparation as well as end product pictures) on social media platforms, e.g. Twitter. This was to highlight to the children that there were many other children taking part in the camp. Parents were advised that they did not need to share identifiable pictures of their children, that it could be progress pictures of the food or the end product. Furthermore, a final collated video of pictures of participants taking part in camp that parents submitted to the research team for inclusion was shared solely among the participants at the end through the parental email. Due to the camp-styled format of the programme, e.g., five daily videos designed to viewed and recipes cooked on consecutive days, the programme ran over the school break in April 2021. The sample size was calculated in relation to perceived cooking competence as the primary outcome. A G*power *a priori* analysis with a significance level of 0.05 indicated that to detect a moderate effect size (0.25), a total sample of 175 participants was necessary [36].

**Table 1:** Camp format and adaptions from original ‘Cook Like A Boss’

Day	Online Camp	Original Camp	Adaption
1	Intro & Italian Day: Introduction, safety & flatbreads	General introductory and knife safety (Chicken chowder & cornbread)	• Moving the day from the order in the original camp, due to removal of pasta, allowing additional time for safety and hygiene • Removal of pasta from scratch due to equipment concerns
2	Chow down: Chicken Chowder and cornbread	Italian food-themed day	• Day swap, due to removal of pasta, allowing additional time for safety and hygiene • Removal of pasta from scratch due to equipment concerns
3	Baking Day: Cake & Cookies	Bakery day: Cake, muffins & cookies	• Removal of muffins due to timings for completing recipes in home environment
4	Vegalicious: Chili Non Carne	‘Plant-based/Vegetable day’: Chili Non Carne	• No adaptions
5	Fakeaway Friday: Honey Chilli Chicken & Dessert	Children’s Choice: Children’s co-creation day – different groups had different recipes, either chicken popcorn, slaw & dessert or Honey Chili Chicken & Dessert	• *No adaption* – Honey Chilli Chicken chosen, due to equipment concerns for popcorn chicken

### Data collection and intervention assessment

Before the intervention began all children completed the ‘pre’ survey via a link sent to the participants’ parents. On the final day of camp, the link for the ‘post’ survey was sent along with the video and participants had a week to complete this survey. Parents were advised that the children should complete the survey individually, however, if they needed assistance with reading or using the technology, this was permitted. To ensure accurate assessment of the children, clarity of the language, question formatting and consideration of children’s attention span are essential [37]. Therefore, validated measures for children were used, questions were presented in an engaging format and the survey was designed to have a 10–15 min duration. The surveys gathered data relating to the children’s demographics, perceived cooking competence, perceived movement competence and wellbeing, the measures included are detailed in Sect. 2.3.1. Process evaluation assessments to understand intervention adherence and fidelity were additionally collected (data not presented here, will be published separately).

#### Survey measures

##### Perceived cooking competence

The validated measure of perceived cooking competence CooC11 [38], was completed by children. CooC11 was an 11-item measure used to assess children aged 8–12 years on their perceived competence of their cooking skills, including skills such as chopping, peeling, weighing ingredients and using an oven. Children were shown illustrations of characters and asked whether they do the skill, if they respond yes, they were then shown two illustrations one of a ‘good’ performance of a skill and one of a ‘poor’ performance. The child was then asked which image they are most like on a five-point Likert scale. The sequence of presentation of ‘good’ and ‘poor’ performance of a skill alternated, and scores were reverse coded where necessary so that a higher score indicated a higher perceived competence. The score for each skill was then summed to create a total cooking competence score, possible scores ranged from 0 to 55.

##### Perceived movement competence

The validated pictorial measure of perceived movement competence [39], was also completed by the participants. It was a 12-item scale (six locomotor skills and six object control skills). Specific movement skills within the scale have elements that would have similar movements within cooking skills. For example, throwing requires arm, wrist and finger movement and control, which could apply to a number of cooking skills such as mashing, spooning and cutting. Another example would include the hand eye coordination and the arm and wrist control required in rolling would be similar movement to inserting food into an oven or scraping down a bowl. Children were shown illustrations of characters performing a skill, with good and poor performance alternated for skills, and required to choose which picture was most like them. Within the chosen picture, children were then asked to further indicate their perceived competence. Scoring was as follows, in the ‘good’ performance option: ‘really good at…’ (Scored four) or ‘pretty good at…’ (Scored three) and for the ‘poor’ performance: ‘sort of good at…’ (Scored two) or ‘not that good at…’ (Scored one). Scores were reverse coded where necessary so that a higher score indicated a higher perceived competence and were summed to create a total perceived movement score, with possible ranges from 12 to 48.

##### Wellbeing

Participants completed The Stirling Children’s Well-being Scale [40], a positively worded scale measuring emotional and psychological well-being in children aged 8–15 years. The measure was a 12-item scale, with 15 items included in total in the formatting and 3 items were not included in the final score as per instructions. The 12-items cover the components of Positive Affect (optimism, relaxation and cheerfulness and satisfying interpersonal relationships) and Positive functioning (competence and clear thinking) and were classified as Positive Emotional State and Positive Outlook. Each item was scored 1 to 5, with possible ranges from 12 to 60.

##### Demographics

Children also completed questions around their age and gender and their location (either Republic of Ireland or Northern Ireland) was established at recruitment. Prior cooking experience was assessed with the proxy question ‘Do you help your mum/dad/other adult making the dinner’ [32, 41]. Children responded on a scale of 1–5, with 1 meaning ‘Always’ and 5 meaning ‘Never,’ scores were reverse coded for analysis so that a higher score indicated more frequent assisting.

### Data analysis

All statistical analysis was conducted using IBM SPSS Version 26. Significance levels were set at 0.05. The demographics of the participants were summarised with descriptive statistics (mean and standard deviation [SD]). Where necessary, prior to data analysis, items were reverse coded so that a higher score showed a more desirable or positive response. Additionally, where required, data was transformed using inverse transformation to ensure the data were normally distributed (for all measure data).

Bivariate correlations using Pearson’s correlation coefficients were used to examine associations between ‘pre’ measures of perceived cooking competence, perceived movement competence and wellbeing, to evaluate the strength of the relationships between these variables. To measure the intervention’s impact, we compared the mean scores of perceived cooking competence, perceived movement competence and wellbeing before and after the intervention using paired-sample t-tests. Effect size of any significant differences was assessed using Cohen’s d, with the meaning of effect size being d (0.01) = very small, d (0.2) = small, d (0.5) = medium, d (0.8) = large, d (1.2) = very large, and d (2.0) = huge [42].

Hierarchical multiple regression modelling was used to determine how much of the variance in the dependent variable (post perceived cooking competence) was accounted for by the predictor variables (demographics and perceived movement competence and wellbeing) while controlling for pre-perceived cooking competence. For regression analyses multicollinearity and collinearity were assessed using multiple methods such as correlations between predictor variables being less than 0.7, cook’s distance not exceeding 1, the standard residuals the minimum and maximum being between − 3 and 3 and the Variance Inflation Factor being below the suggested critical value of 10 [43]. In addition, all predictor variables should correlate with the outcome above 0.3. Furthermore, autocorrelation was assessed using the Durbin-Watson test, with a value of 2 indicating no autocorrelation.

## Results

248 participants fully completed the pre-survey. However, there was matched pre/post surveys for 210 participants that were included in the analysis. The difference is accounted for by different children in the same household completing the pre/post surveys and children retention. The baseline demographics of the participants are summarised in Table 2. The majority of participants were girls (75.7%) and from the Republic of Ireland (64.3%). There was an even spread of children across the different ages. A small minority of the sample reported cooking on a regular basis ‘always or often’ (12.7%).

**Table 2:** Sociodemographic characteristics of the ‘Cook Like A Boss’ participants

Characteristic		
*Total (N)*	*210*	
***Participants***	*Mean*	*SD*
Age (years)	10.52	1.10
	*N*	*%*
***Gender***		
Boy	51	24.3
Girl	159	75.7
***Location***		
Northern Ireland	75	35.7
Republic of Ireland	135	64.3
***Age***		
9 years old	47	22.4
10 years old	59	28.1
11 years old	51	24.3
12 years old	53	25.2
Cooking Experience*		
Always	3	1.4
Often	24	11.3
Sometimes	128	60.4
Rarely	41	19.3
Never	16	7.5

*Assisting a parent/guardian with dinner preparation

### Associations between perceived cooking competence, perceived movement competence and wellbeing pre-intervention

A significant positive correlation was shown between perceived cooking competence and perceived movement competence (r = 0.30, *P* < 0.001), as well as with wellbeing (r = 0.16, *P* = 0.019). A significant positive correlation was found between perceived movement competence and wellbeing (r = 0.35, *P* < 0.001).

### Intervention effect on outcome variables

Children’s perceived cooking competence (*P* < 0.001), perceived movement competence (*P* < 0.001) and wellbeing (*P* = 0.013) all significantly increased from pre to post intervention (Table 3). A medium to large effect size was found for perceived cooking competence, a small to medium effect size was seen for perceived movement competence and a small effect size was found for wellbeing.

**Table 3:** Intervention effect on perceived cooking competence, perceived movement competence and wellbeing using paired samples t-tests

Variable			Mean (SD)		t	P	Cohen’s d
	**Original Possible Ranges**	**Transformed ranges**	**Pre-Intervention**	**Post-Intervention**			
**Perceived Cooking Competence**	0–55	0.21–65.14	31.58 (11.96)	37.48 (9.84)	-9.87	< 0.001	-0.68
**Perceived Movement Competence**	12–48	23.91–53.23	37.98 (5.40)	39.48 (5.82)	-5.16	< 0.001	-0.36
**Wellbeing**	12–60	30.40-62.76	46.64 (6.00)	47.42 (6.42)	-2.50	0.013	-0.17

### Predictors of post programme perceived cooking competence

Table 4 displays the results of a hierarchical multiple analysis in predicting post programme perceived cooking competence. No multicollinearity or collinearity were found. And the Durbin-Watson was deemed acceptable at 2.09, indicating no auto-correlation was detected. Three potential predictor variables, one demographic (gender), pre-intervention wellbeing and post-intervention perceived movement competence were removed from the model as they did not sufficiently correlate with the dependent variable. The baseline model controlled for the participants pre programme perceived cooking competence, a potential predictor for post programme perceived cooking competence. This variable accounted for 49% of the variance in post programme perceived cooking competence, with a significant independent contribution (*P* < 0.001). Due to the accumulative nature of the models, models 1 and 2 control for initial variables and model 3 tests the impact of perceived movement competence and wellbeing on post programme perceived cooking competence. Model 2 included the participants’ age and prior cooking experience, these variables accounted for an additional 1% of the variance (*P* < 0.05). Model 3 included participants’ pre perceived movement competence and post wellbeing and accounted for a further significant 7% of the variance (*P* < 0.001) being explained. Each model explained a significant amount of variance (*P* < 0.05), with the final model (3) explaining 57% of the total variance in participants post programme perceived cooking competence. The final significant predictors of post programme perceived cooking competence included: *pre programme perceived cooking competence*, post *wellbeing*, *age* and pre *perceived movement competence*.

**Table 4:** Results of Hierarchical multiple regression predicting post programme perceived cooking competence

Variables	Model 1		Model 2		Model 3	
	**B (SE)**	**Β**	**B (SE)**	**β**	**B (SE)**	**β**
Pre CooC11	0.577 (0.041)	0.700***	0.529 (0.050)	0.643***	0.457 (0.049)	0.555***
Age			1.173 (0.458)	0.131*	1.593 (0.432)	0.178***
Cooking experience			0.478 (0.720)	0.039	0.493 (0.686)	0.040
Pre movement competence					0.242 (0.094)	0.133*
Post Wellbeing					0.309 (0.075)	0.204***
**F**	200.40***		70.59***		55.71***	
**Adjusted R** ^**2**^	0.488***		0.500*		0.567***	

*P < 0.05, **P < 0.01, ***P < 0.001

## Discussion

‘Cook Like A Boss’ Online is an innovative and effective virtual cooking camp for children, that was implemented during the COVID-19 pandemic and adapted from a theory-based co-created in-person camp. It is the first children’s culinary intervention to assess perceived cooking competence, perceived movement competence and wellbeing in one study and provides essential initial evidence of the associations between these variables.

‘Cook Like A Boss’ [32] was the first intervention guided by a cooking education model [33], that included co-creation with the children participating and was underpinned by Social Learning Theory and Experiential Learning Theory. This intervention was adapted to be delivered in a virtual format due to the COVID-19 pandemic. The adaption from an in-person format to virtual one, while the largest adaption, it has merit as video can be a useful tool for teaching cooking skills [44]. Additionally, before the pandemic there had been a reduction in children learning cooking skills in the home environment due to time pressures and fears [13], yet during the pandemic there was an increased willingness to involve children in cooking activities [11]. The programme provided a structured opportunity for parents to include children in cooking activities that were age-appropriate, as the recipes had been deconstructed to match their underlying age-related motor skills [19], helping to reduce parental fears around what age children should be performing certain skills, e.g. chopping with a knife. Furthermore, children, adolescents and parents prefer to receive lifestyle behaviour-change information via the internet through their devices, such as phones, computers and tablets in comparison to traditional in-person methods [45, 46]. In addition, e-health interventions have been shown to be an effective method for child health treatments [47]. All suggesting that an e-health approach was an optimal and valid adaption to the programme, albeit being a necessity during the COVID-19 pandemic.

The principles of ELT and SLT remained within the programme, gaining a hands-on experience to develop an initial interest and enable lifelong learning (ELT), mastery experiences and reducing individuals’ stress reactions towards challenging situations (SLT). Similar to the original camp, children took part in mastery experiences, preparing recipes they had never been exposed to or cooked before repeatedly throughout the camp. However, notably to stay safe, children participated at home. While some children may have had siblings joining in the cooking activities, and while the research team attempted to generate a level of social connectedness through parental engagement on social media and sharing an end video of all children participating with them, this level of social connectedness was reduced in comparison to the original camp. This reduction in social models (some level provided through the virtual videos) and social persuasion, two key elements for increasing perceived competence according to SLT, may contribute towards a smaller effect size of the virtual ‘Cook Like A Boss’ camp in comparison to the original camp on perceived cooking competence [32]. This highlights a significant aspect for consideration for future e-health interventions, the role of ‘social’ (connectedness, peer modelling, mentor modelling). The current intervention is an example of optimised conditions, with a captive audience in want of virtual activities, and while a general lack of social interactions may have had some influence, it was not able to match the effect size of the in-person camp for improving perceived cooking competence. The original camp’s process evaluations highlighted the importance of peers and facilitators to the children, while the virtual camp was able to improve perceived competence; a hybrid model is an approach that could be considered for future e-health interventions for children.

As perceived competence is a motivator for performing a behaviour [31], it is essential to explore the relationships between differing but related perceived competences. Fine motor skills (the use of small muscles) and gross motor skills (the use of larger muscles) are involved in movements that require the functioning of the extremities [48], and have been proposed as essential to understanding the performance of cooking skills and what cooking skills children should be able to perform at different ages [19]. Children with higher levels of perceived movement competence have also been shown to participate in a greater amount of physical activity [49]. With declining levels of physical activity among children [50], it is important to understand different methods that can be utilized to improve perceived movement competence. This study provided initial evidence of the positive associations between perceived cooking competence, perceived movement competence and wellbeing. It may also indicate that improving one of the variables may have a positive influence on the other and the regression analysis indicating that pre intervention perceived movement competence is a significant predictor for post perceived cooking competence. Physical activity is associated with positive mental health and wellbeing [23, 24, 51–53]. While cooking has been linked to wellbeing in adults and adolescents [25, 26], with a proposed mechanism for this link being the physical movement interacting with neurobiological pathways such as dopaminergic and serotonergic which then reduces distress [27]. This research highlights how increasing perceived competence in these areas may also have an impact on wellbeing. One experiential cooking intervention in children and adolescents in the USA found a modest yet significant increase in wellbeing using a validated measure after a six-week programme [54], a similar result to that was found in the current study. Differences in sample ages, location, mode of delivery with a similar result may indicate the importance of the actual experiential cooking aspects for wellbeing. Additionally, the small significant increase in wellbeing that occurred post programme occurred despite the highly stressful global pandemic that caused anxiety in children [7]. This initial evidence supports the need for further research into the use of cooking, in terms of both perceived competence and the tangible act of cooking, and wellbeing in children.

After taking part in the short duration virtual cooking camp, significant increases in perceived cooking competence, perceived movement competence and wellbeing were evidenced. As the intervention was camp-styled, e.g. designed for consecutive days, it influenced the short duration of the programme. However, future research should investigate whether a longer duration of cooking classes or the classes spaced apart or repeated participation could attain greater improvements in perceived competences and wellbeing. Furthermore, while this research highlighted the relationship of cooking with perceived movement competence, future studies should explore cooking as a tool for increasing actual motor competence in children, as research indicated children are not developing their fine motor skills at a normative rate [55]. As cooking is a life skill, it is routinely used in occupational therapy with patients following stroke or Traumatic Brain Injuries and people with disabilities to enhance their functional capacity [56–58], research should investigate whether it could be used as a beneficial tool in general populations to enhance their development. The use of technology to deliver the programme is intriguing, as technology has been implicated as a potential mechanism that is changing the trajectory of children’s fine motor development [55, 59]. However, as technology is so ingrained in children’s lives [60, 61], this concept of using technology to encourage an activity that could potentially improve children’s fine motor skills could be a mechanism to integrate technology with fine motor promoting activities.

Finally, the hierarchical multiple regression indicated that initial perceived cooking competence explained the largest amount of variance in perceived competence after the programme. This highlights the need to further understand what influences children’s primary perceived competence. For example, prior experience was not a significant predictor of perceived cooking competence at the end, however it may have a role in their beginning perceived competence. Additionally, parental influence on children is another aspect for consideration as seen in education where parental education can influence children’s educational attainment [62], and their cooking confidence can influence a child’s intake of ultra-processed foods [63]. By extension, it is plausible that parental cooking confidence could affect children’s perceived cooking competence and warrants further research.

Perceived movement competence and wellbeing remained significant predictors and explained a significant variance for perceived cooking competence after the programme as well. This, along with the other findings, shows that these concepts are highly interrelated and further research is required to understand their associations. Additionally, age remained a significant predictor in the final model. While the recipes were deconstructed and mapped back to age-appropriate, cooking skills in line with Dean et al. [19] to ensure the content was achievable for all children, differences in actual motor coordination between the children at different ages [55] may have impacted the level at which the child could complete the skill. This in turn, may have then influenced their perceived cooking competence.

This research has shown that cooking is a complex activity that can have an impact on wider components than the traditional focus of improving diet quality and dietary intake. It has the potential to be a multi-focused solution for not only improving diet quality and perceived cooking competence, but movement and wellbeing as well.

### Strengths and Limitations

Key strengths of the current study are the minor adaptions (albeit necessary due to the nature of the pandemic) to the content of an effective children’s cooking programme. Additionally, a criticism of children’s cooking interventions and wider nutrition interventions with children in general, is a lack of validated instruments for measuring outcomes [16, 64, 65]. This research uses validated measurement tools for all its outcomes, which is a significant strength. Furthermore, another weakness in research design in this area is a lack of power calculations [18]. This study was sufficiently powered. Additionally, this programme was designed as an outreach programme, to provide support and an activity for children during the COVID-19 pandemic.

While there are a number of strengths to this research, some limitations must be considered and in turn provide areas for improvement for future studies. While it was deemed unethical in light of the unprecedented situation, the main limitation to this this research was the lack of a control group. The online intervention was adapted from an effective programme assessed with a control group [32], however it would be beneficial to assess the findings using a control group. Additionally, while validated measures were used in this study, it is worth noting that the perceived movement measure was initially validated in younger ages, however it has since been widely used in age groups similar to the group in the current study. It has also been suggested that The Stirling Wellbeing measure is best utilized over a few weeks for assessment. However, it’s temporal stability was assessed and shown to be stable over a one-week period [40], which would suggest changes found over a week are not due to unreliable measurement. A similar limitation found in this study as in the original camp intervention, is the gender imbalance. While this camp was also filled on a ‘first come, first served’ basis, with additional funding secured to increase capacity due to interest, the majority of participants were girls. This may be due to societal norms around cooking still being perceived as a woman’s responsibility [66–68]. Future research could actively target boys for recruitment to cooking interventions, especially considering the fine motor skills required for cooking and boys underperforming in fine motor skills in comparison to girls at young ages [69].

## Conclusion

The ‘Cook Like A Boss’ Online intervention was an adapted virtual outreach programme delivered during the COVID-19 pandemic. It was the first children’s cooking intervention to assess children’s perceived cooking competence, perceived movement competence and wellbeing, and highlight the associations between these variables as well as being effective in their improvement. While traditionally, cooking interventions target diet quality and dietary intake, this research shows the potential for cooking to be used as a mechanism for targeting improvements in wider aspects of lifestyle such as movement and wellbeing.

## Supplementary information

Additional file 1. TIDieR-Checklist-Word, Cook Like A Boss Online

## Data Availability

The datasets generated and/or analysed during the current study are not publicly available due to ethical approval procedures.

## Abbreviations

ELT: Experiential Learning Theory
SLT: Social Learning Theory
